# Identification of SALL4 Expressing Islet-1+ Cardiovascular Progenitor Cell Clones

**DOI:** 10.3390/ijms24021780

**Published:** 2023-01-16

**Authors:** Andrea Monteon, Lorelei Hughes, Victor Camberos, Mary Kearns-Jonker

**Affiliations:** Department of Pathology and Human Anatomy, Loma Linda University School of Medicine, Loma Linda, CA 92350, USA

**Keywords:** Islet-1, cardiovascular progenitor cells, SALL4

## Abstract

The utilization of cardiac progenitor cells (CPCs) has been shown to induce favorable regenerative effects. While there are various populations of endogenous CPCs in the heart, there is no consensus regarding which population is ideal for cell-based regenerative therapy. Early-stage progenitor cells can be differentiated into all cardiovascular lineages, including cardiomyocytes and endothelial cells. Identifying an Islet-1+ (Isl-1+) early-stage progenitor population with enhanced stemness, multipotency and differentiation potential would be beneficial for the development of novel regenerative therapies. Here, we investigated the transcriptome of human neonatal Isl-1+ CPCs. Isl-1+ human neonatal CPCs exhibit enhanced stemness properties and were found to express Spalt-like transcription factor 4 (SALL4). SALL4 plays a role in embryonic development as well as proliferation and expansion of hematopoietic progenitor cells. SALL4, SOX2, EpCAM and TBX5 are co-expressed in the majority of Isl-1+ clones isolated from neonatal patients. The pre-mesendodermal transcript TFAP2C was identified in select Isl-1, SALL4, SOX2, EpCAM and TBX5 expressing clones. The ability to isolate and expand pre-mesendodermal stage cells from human patients is a novel finding that holds potential value for applications in regenerative medicine.

## 1. Introduction

Isl-1 is a well-recognized marker of early-stage cardiac stem cells [1]. This transcription factor identifies a distinct population of undifferentiated progenitors capable of generating cardiomyocytes, vascular smooth muscle, and endothelial cells both in vitro and in vivo as demonstrated by multiple approaches [2]. Islet-1 is expressed in progenitors of both primary and secondary heart fields, according to fate mapping [3]. Transcriptomic studies have identified Isl-1 expression at the mesendoderm stage of cardiac development. SALL4 is a marker expressed very early at the pluripotent stage of development and remains to be expressed at the pre-mesendoderm stage [4]. Stage-specific CPC development has been studied in human embryonic stem cells (hESCs) and induced pluripotent stem cells (iPSCS); however, little is known about the transcriptome of neonatal CPCs isolated from cardiovascular tissue derived from human neonates. We demonstrate here that SALL4 and pre-mesendodermal transcripts are expressed in human neonatal Isl-1+ CPC clonal populations isolated from the cardiovascular tissue of 8-day old neonates. Our study shows that neonatal CPCs represent a patient-derived and enriched source of unique stem cell populations, including Isl-1+ CPCs at the pre-mesendoderm stage of development which can be characterized by cells expressing SALL4 and TFAP2C.

## 2. Results

### 2.1. Islet-1+ Cardiovascular Progenitor Cell Clones Express Early-Stage Markers

**Figure 1:**
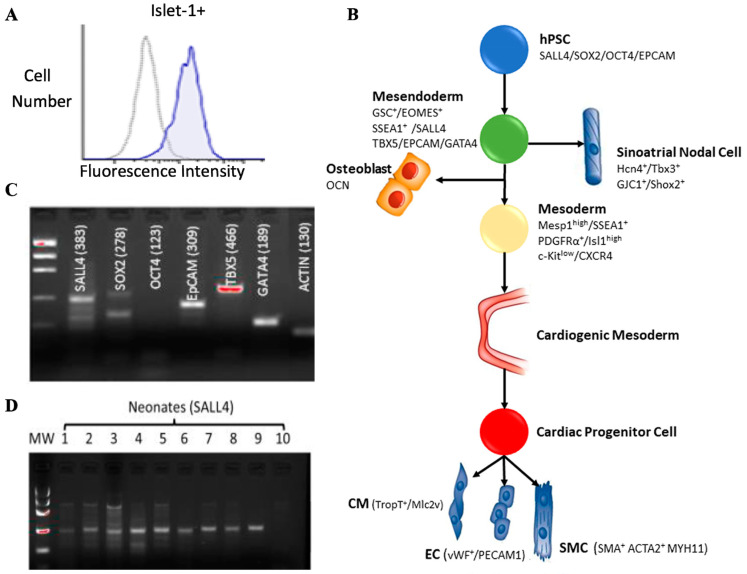
Isl-1+ neonatal CPCs express early-stage transcripts. Flow cytometry confirmed expression of Isl-1 in human neonatal CPC clones (**A**). Stemness associated transcripts appear early in cardiovascular development (**B**). PCR products were run on an agarose gel, transcripts for SALL4, SOX2, EpCAM, and TBX5 were co-expressed on a representative Isl-1+ neonatal CPC clone (**C**). PCR products were run on an agarose gel showing SALL4 expression in nine out of ten Isl-1+ neonatal CPC clones (**D**).

Early-stage markers of cardiovascular development were identified in Islet-1+ neonatal CPC clones derived from human patient tissue. Islet-1+ is expressed in CPC clones as demonstrated by flow cytometry (Figure 1A). Specific markers appear at distinct points during cardiovascular development (Figure 1B). This data suggests that a multipotent Islet-1+ cardiac progenitor that gives rise to cardiomyocytes, smooth muscle, endothelial cells in all chambers of the heart exists. Using RT-PCR and gel electrophoresis, early-stage markers such as SALL4, SOX2, OCT4, EpCAM, TBX5 and GATA4 were expressed in a representative neonatal Islet-1+ CPC clone (Figure 1C). Gene specific primers were used to assess the expression of SALL4 via RT-PCR in ten Islet-1+ neonatal CPC clones. Nine of the ten CPC clones tested expressed SALL4 (Figure 1D).

### 2.2. Islet-1+ Neonatal CPC Clones Express Characteristics of Enhanced Stemness

**Figure 2:**
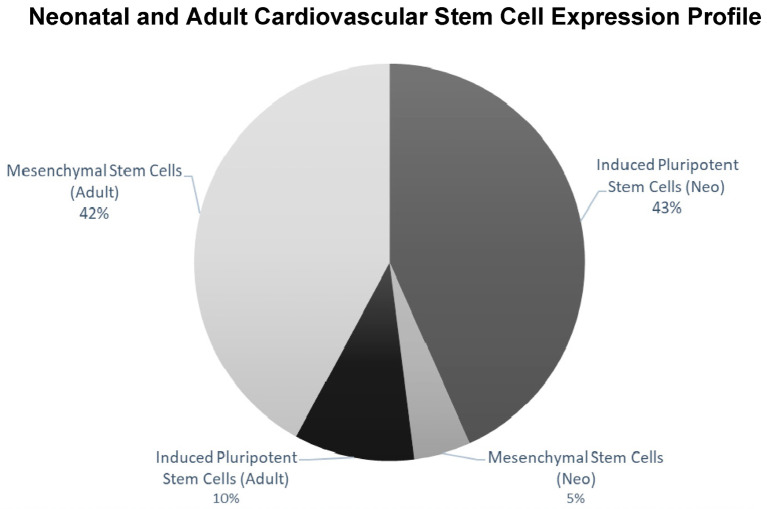
StemChecker demonstrates differences when comparing the iPSC and MSC expression profile for Isl-1+ neonatal and adult CPCs. Isl-1+ neonatal CPC (N = 3) transcriptome had the greatest number of transcripts corresponding to iPSCs. Isl-1+ adult CPC (N = 3) transcriptome had the greatest number of transcripts corresponding to MSCs.

RNA-Seq based transcriptomics was used to identify the expression profile and stemness features within the neonatal and adult Isl-1+ CPC population. Using StemChecker “http://stemchecker.sysbiolab.eu/as (accessed on 12 December 2021)”, the Isl-1+ neonatal CPC clones (N = 3) presented a gene expression profile most similar to induced pluripotent stem cells (*p*-value = 0.024). In contrast, the transcriptome of the Isl-1+ adult CPC clones (N = 3) presented a gene expression profile most similar to mesenchymal stem cells (MSC) (*p*-value 2.01 × 10^−6^) (Figure 2).

### 2.3. Transcriptomic Profiling Reveals SALL4 Interacts with Pluripotency Factors

**Figure 3:**
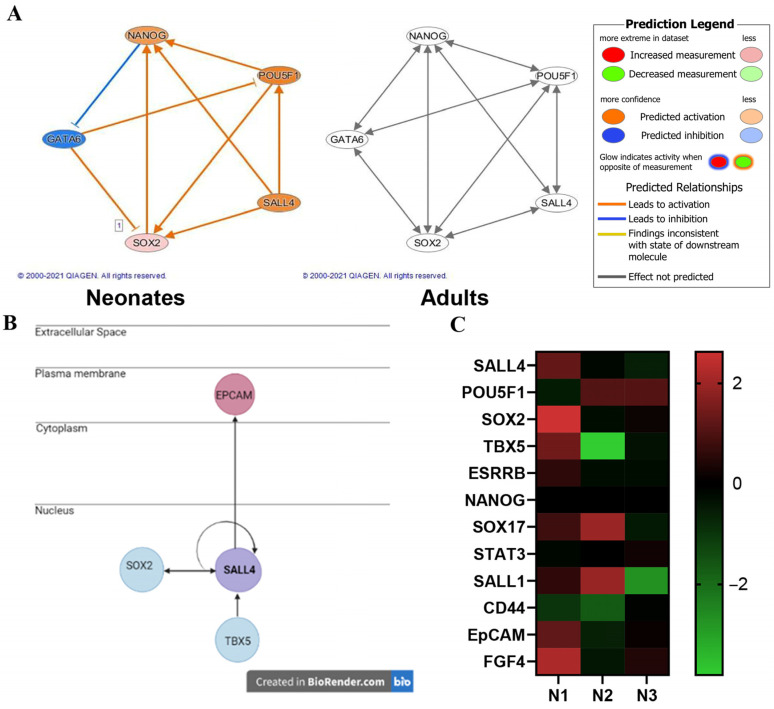
Transcriptomics reveals SALL4 interacts with pluripotency factors in Isl-1+ neonatal CPCs via a mechanistic network of regulators. The predicted activation of a network of genes in neonatal Isl-1+ CPCs (N = 3) (**left**) was not identified in adult Isl-1+ CPCs (N = 3) (**right**) using the IPA tool: molecule activity predictor (**A**). Genes associated with SALL4 (**B**). Heatmap of genes associated with SALL4 that are expressed in three neonatal CPC clones identified as N1, N2 and N3 (**C**).

Gene expression profiles of the Isl-1+ neonatal and adult CPC clonal populations were compared to one another using Ingenuity Pathway Analysis (IPA). A fold change cutoff of 2.0 was applied to focus on statistically significant gene expression changes when comparing each age group. The neonatal population (N = 3) consisted of 2673 transcripts and the adult population (N = 3) consisted of 1381 transcripts. Transcript sequence information was used to identify mechanistic networks. A mechanistic network was identified in the neonatal population data containing key stemness genes: SOX2, OCT4, and Nanog. In addition, Gata 6, an early-stage differentiation gene was found in the network. This mechanistic network was not identified in the adult CPC population. Stemness gene SALL4 interacts with SOX2, OCT4 and Nanog; therefore, SALL4 was added to the network using the IPA grow tool. IPA path explorer was used to determine the connection between SALL4 and the stemness genes. The connections are based on the IPA knowledge base which is curated from published experimental data. The molecule activity predictor function was applied to the network to identify predicted activation and inhibition of the molecules as determined by the z-score. Figure 3A shows that this mechanistic network is predicted to be activated in the neonatal population while the network shows no significant activation in the adult population. SALL4 interacts with SOX2, EpCAM and TBX5 (Figure 3B). SALL4 increases the expression of SOX2. Activation of SALL4 is predicted to be involved in upregulation of EpCAM mRNA. Neonate 1 (N1) demonstrated the greatest correlation of genes associated with SALL4 activation/function compared to Neonates 2 and 3 (Figure 3C).

### 2.4. Transcriptomic Profiling Elucidates Pathway and Stage-Specific Differences between Neonatal CPC Clones

**Figure 4:**
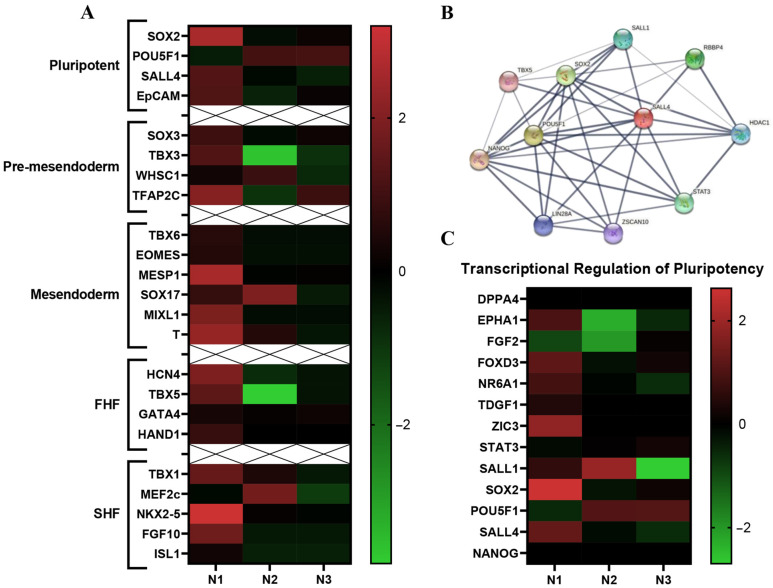
Stage specific differences within the Isl-1+ neonatal CPC population. Heatmap of markers that identify stages (pluripotent, pre-mesendoderm, mesendoderm, FHF, SHF) of CPC development between the individual neonatal Isl-1+ CPC clones. Neonate 1 expresses the highest number of early-stage transcripts (**A**). String ID of genes that play a role in the transcriptional regulation of pluripotency pathway (**B**). Neonate 1 expresses the highest number of genes associated with the transcriptional regulation of pluripotency pathway (**C**).

Cardiogenesis begins at the start of gastrulation. Specifically, epiblast cells ingress through the primitive streak to form the mesendoderm and subsequently mesoderm [5]. Stage specific gene expression during cardiogenesis is used to identify differentiation steps in development. Comparing previously published stage-specific RNA-seq transcriptomic data to our RNA-seq Isl-1+ neonatal CPC transcriptomic data yields stage specific differences in gene expression within the Isl-1+ neonatal CPC clones (Figure 4A). Stages of CPC development are: Pluripotent/epiblast, pre-mesendoderm, mesendoderm, FHF and SHF. Each individual Isl-1+ neonatal clone showed differences in expression level. Specifically, the heatmap (Figure 4A) shows that N1 expresses the highest number of genes correlated to all CPC developmental stages when compared with N2 and N3.

Using the comparative transcriptomic data of three individual’s patient-derived Isl-1+ neonatal CPC clones, all fold change values were entered into IPA software to identify the differences and similarities within the neonatal population. Each Isl-1+ neonatal clone showed differences in pathway-specific transcript expression levels when comparing Neonate 1, Neonate 2 and Neonate 3. Specifically, the transcriptional regulation of pluripotency pathway was identified. To understand how genes within this pathway interact, a String ID pathway analysis was performed (Figure 4B). String ID allows for the visualization of direct and indirect protein-protein interactions by implementing well known classification systems for enrichment analysis. Transcriptomic analysis revealed genes within the pathway to be most highly expressed in the clone isolated from Neonate 1. Although transcripts identified within the Transcriptional Regulation of Pluripotent Stem Cell pathway were identified in each of the three Isl-1+ neonatal CPC clones, Neonate 1 expressed the highest levels (Figure 4C).

### 2.5. Select Islet-1+ CPC Clones Express TFAP2C

**Figure 5:**
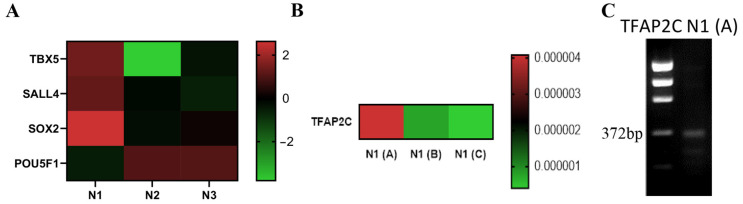
TFAP2C reveals a subpopulation of Isl-1, SALL4, TBX5, EPCAM, SOX2 expressing neonatal CPC clones. Heatmap visualization generated from Isl-1+ neonatal RNA-Seq data showing differentially expressed genes in Neonate 1, Neonate 2 and Neonate 3 CPC clones (**A**). Heatmap visualization of RT-PCR analysis of TFAP2C mRNA levels in three neonatal CPC clones derived from 8-day old patients. These clones are represented as Neonate 1 (N1A), Neonate 1 (N1B) and Neonate 1 (N1C). RT-PCR was performed on cDNA using gene specific primers (**B**). Gel image of TFAP2C (372 bp) mRNA expression in N1 (**A**) Isl-1+ neonatal CPC clone expression as detected by RT-PCR (**C**).

SOX2, EpCAM and TBX5 expression was validated by transcriptomics and RT-qPCR in the Islet-1+ CPC clone isolated from Neonate 1. A gene specific primer for transcription factor TFAP2C was then used to further define the developmental stage of the SALL4, SOX2, TBX5, and EpCAM expressing Isl-1+ CPC clones. The clone isolated from Neonate 1 demonstrated expression of TFAP2C, consistent with that of an early mesendodermal stage cell. Therefore, we sought to determine whether or not all day 8 CPC clones have a gene expression profile similar to Neonate 1. We compared three independent CPC clones identified from different 8-day old neonates and found that while all day 8 CPC clones expressed transcripts encoding SALL4, SOX2, TBX5, and EpCAM, TFAP2C was not expressed in all of these clones. TFAP2C is expressed on day 2 of directed cardiomyocyte differentiation, with day 2 corresponding to early mesoderm. A heatmap derived from RT-PCR analysis revealed that Neonate 1 (labeled N1A) in Figure 5) expresses TFAP2C while Neonate 1(B) and Neonate 1(C) clonal samples do not, thereby confirming that the day 8 Isl-1+ neonatal CPCs are not all similar and can be sub-staged (Figure 5C). Gel electrophoresis of the RT-PCR product confirmed the size of the amplified product. TFAP2C expression confirms that Neonate 1(A) represents the earliest cardiac progenitor cell clone that has been isolated from neonatal human cardiovascular patient samples. TFAP2C was expressed in 6% of all neonatal CPC clones examined in our study.

## 3. Discussion

In the present study, SALL4 -expressing clonal populations of Isl-1+ CPCs were isolated from human neonatal cardiovascular tissue. TFAP2C was expressed within a subpopulation of cells that exist at the pre-mesendodermal stage of development in newborns. TFAP2C drives mesendoderm competence and shifts the developmental trajectory toward the pluripotent state [6]. The enhanced stemness of the transcriptional profile that characterizes cells residing in the cardiac tissue of neonates may contribute to their well-documented regenerative ability. This study identifies the earliest pre-mesendodermal cells that reside in, and can be isolated from, the neonatal heart.

There is limited evidence identifying the functional differences that distinguish stage specific cardiac progenitor cells, in part due to the challenge of expanding clonal populations of these cells. It has been reported that early-stage SSEA-1+/Isl-1+ CPCs derived from Rhesus ESCs and transplanted in an allogeneic setting reduce scar tissue size and differentiate into ventricular myocytes with no evidence of teratoma formation [7]. These early CPCs go through the mesendodermal stage of development, segregate into FHF and SHF derivatives and further give rise to the 3 main cardiac lineages of the heart under induced cardiac differentiation. The absence of inappropriate differentiation in vivo suggests that early-stage cells take cues from the cardiac environment to achieve lineage specific differentiation. Lineage tracing studies in mice showed that development of the heart and hindlimb is regulated by Isl-1 [8]. Therefore, Isl-1 may mark a subset of early-stage progenitors that differentiate along a common heart/hindlimb pathway of development. According to Akiyama et al., SALL4 plays a critical role at early steps of limb development [9]. Specifically, inactivation of SALL4 in the mesendoderm before limb outgrowth causes defects of proximal-anterior skeletal elements specifically in the hindlimbs [10]. While Isl-1 has been elucidated in cardiac and hindlimb progenitor cells, SALL4 has yet to be examined in CPCs.

Transcriptomics provides additional insight into developmental processes. Stavish et al., found that the exit from pluripotency involves intermediates that retain pluripotency while simultaneously exhibiting lineage-bias [11]. Specifically, a sub-state of human pluripotent stem cells expresses the early endoderm marker GATA 6. GATA 6 positive cells are able to regenerate long-term pluripotent cultures and differentiate toward the endodermal lineage [12]. Sub-states that co-express pluripotency and differentiation markers represent differentiation intermediates and exist as transient states during development. Other studies using trajectory mapping and RNA seq analysis have revealed branch points in hESC cardiac differentiation which result in a continuous pluripotent to cardiomyocyte differentiation trajectory [13]. Multiple lineages including cardiac mesoderm, endoderm-like cells, and fibroblast like cells branch off of a pluripotent cluster. Interestingly, a global transcriptional dynamics analysis across time showed that gene expression differed significantly between days 3 and 6 suggesting that up until day 6, cells rapidly progress through transient and distinct cellular states. Mononen et al., published transcriptomic data showing that the pluripotent cluster, cardiac mesoderm cluster, and endoderm cluster all expressed SALL4. Taken together, these studies support the concept that the expression of markers of mesendoderm as well as pluripotency are expressed in an intermediate cell stage that is very early along that cardiovascular path.

SALL4 co-expressing Isl-1+ neonatal CPCs express transcripts which are involved early in embryonic development such as TBX5, SOX2, and EpCAM. Additionally, these transcripts are also expressed early along the heart/hindlimb pathway. A subpopulation co-expressing these genes and TFAP2C may reside in a stage of development which is earlier than mesendoderm. Using microarray analysis, Tanimura et al. found that SALL4 and SOX2 bind to the promoter region of the same genes in ESCs [14]. This suggests that the SALL4 and SOX2 overlapping gene set is enriched for genes involved in maintaining pluripotency. SALL4 plays a role in various tissues and organs during embryonic development. Disruption of SALL4 in ESCs results in early embryo defects and lethality during peri-implantation [15]. Additionally, heterozygous disruption of the SALL4 allele leads to multi-organ malformations including limb and heart defects [16]. Specifically, TBX5 regulates SALL4 expression in the developing heart and forelimb and interacts with SALL4 to synergistically regulate downstream gene expression [17]. Mesodermal and cardiac gene expression in OCT4-induced human mesendodermal, mesodermal and neighboring cells was monitored by lineage tracing analysis in the presence or absence of SALL4 and it was shown that expression of genes that play a role in cardiac development such as MESP1, MYOCD, GATA4, TBX5, Isl-1 and Mef2c were induced to a lesser extent in the absence of SALL4 [18]. This suggests that SALL4/TBX5/SOX2/EpCAM co-expressing Isl-1+ cardiac progenitors reside early in embryonic development. The prevalence of SALL4, SOX2, EpCAM, and TBX5 expression in the majority of Isl-1+ neonatal CPC clones led us to identify TFAP2C as a transcript which could further sub-stage these cells. Transcriptomics data published by Churko et al., demonstrated that TFAP2C is expressed transiently at day 2 of directed cardiomyocyte differentiation from hiPSCs [19]. Additionally, Valcourt et al., revealed that overexpression of TFAP2C hindered movement along the hESC developmental trajectory, shifting cells toward the pluripotent state while remaining poised for mesendoderm specification [20]. TFAP2C was expressed in very few clones and only in those isolated from 8-day old neonatal cardiovascular tissue samples. This suggests that not all 8-day old neonatal Isl-1+ CPC clones are at the same developmental trajectory.

Isl-1+ neonatal CPCs exhibit a transcriptomic profile that is similar to iPSCs, according to StemChecker. RNAseq analysis identified the transcriptional regulation of pluripotency pathway as significant which includes transcription factor SALL4. This pathway activates expression of other pluripotency associated factors while repressing lineage specific genes [21]. This component of the transcriptome accounts for how cells can sustain self-renewal and pluripotency while remaining poised for differentiation. In a transplantation setting for cardiovascular repair, early-stage cells that express pluripotency and lineage biased markers are not rejected after transplantation [22]. The results reported here provide new insight into a unique resource of early-stage Isl-1+ CPCs which differ from those reported in studies using differentiated iPSCs because these cells can be isolated from discarded human cardiovascular tissue. The ability to isolate and expand these early-stage cells for experimental and clinical applications may prove to be valuable as the search for an optimal cell source for cardiac regenerative therapy continues.

## 4. Methods

### 4.1. Ethics Statement and Maintenance of Cardiac Progenitor Cells

The Institutional Review Board of Loma Linda University approved the protocol for use of tissue that was discarded during cardiovascular surgery, without identifiable private information, for this study with a waiver of informed consent.

### 4.2. Cardiac Progenitor Cell Expansion

CPC clones were isolated from cardiac tissue of five neonatal (1 day–1 month) and five adult (57–72 years) patients undergoing cardiovascular surgery as previously described by our laboratory [23]. Briefly, discarded cardiac tissue was cut into small clumps (~1.0 mm^3^) then enzymatically digested using collagenase at a working concentration of 1.0 mg/mL for 2 h at 37 degrees Celsius. The resulting solution was then passed through a 40-um cell strainer. Cells were cloned in a 96 well plate by limiting dilution to a final concentration of 0.8 cells per well and were screened to identify Isl-1+ clones. These cells were expanded in media which contained 10% fetal bovine serum (Thermo Scientific, Waltham, MA, USA), 100 µg/mL Penicillin-Streptomycin (Life Technologies, Carlsbad, CA, USA), 1.0% minimum essential medium non-essential amino acids solution (Cat no. 11120052, Life Technologies, Carlsbad, CA, USA), and 22% endothelial cell growth media (Lonza, Basel, Switzerland) in Medium 199 (Life Technologies, Carlsbad, CA, USA) [23]. Twenty clones were expanded for use in the RT-PCR experiments described here and an additional six clones were analyzed by RNA sequencing

### 4.3. Purification of Total RNA

Total RNA was purified from individual CPC clones stored in RNA Protect (Qiagen, Valencia, CA, USA) and Qiazol Reagent (Qiagen, Valencia, CA, USA). 1 mL of RNA protect or 700 µL of Qiazol was used to lyse the cells before being centrifuged though the provided RNeasy spin column followed by a series of washes with provided buffers. RNA was eluted with RNAse-free water and optical density of RNA was read using Nanodrop 2000 to get the concentration of RNA. RNA was run on a 1% agarose gel to assess purity. First Strand cDNA synthesis was performed from 2 µg total RNA using Superscript III Reverse Transcriptase (Invitrogen, Waltham, MA, USA).

### 4.4. RNA Sequencing

RNA samples isolated from neonatal and adult Isl-1+ CPC clones were sent to PrimBio Research Institute for RNA-Sequencing (Exton, PA, USA). rRNA was removed from the total RNA sample using an rRNA removal kit from Illumina (San Diego, CA, USA) (cat# MRZG12324). Sequencing libraries were assembled with an Ion Total RNA-Seq Kit v2 from Thermo Fisher (Waltham, MA, USA) (Cat# 4479789). Nucleic acid binding beads from Ambion (Austin, TX, USA) were used to purify the cDNA library (Cat# 4479681) prior to PCR amplification. Agilent dsDNA High Sensitivity kit was used to determine the quality of the library (Agilent, Santa Clara, CA, USA). The samples were enriched via an Ion OneTouch ES instrument and an Ion PI™ Hi-Q™ OT2 200 Kit (Thermo Fisher, Waltham, MA, USA). Sequencing was performed using an Ion Proton sequencer (Thermo Fisher, Waltham, MA, USA) and a species-specific protocol for our samples. Next, sequence files were aligned to the human genome and quality was determined using the Strand NGS program. Normalization and quantification of the aligned reads were performed using the Deseq algorithm within the Strand NGS program. The Audic–Claverie test and the Benjamini–Hochberg correction test were used for statistical analysis. Significance was determined using a 2.0-fold change minimum cutoff.

### 4.5. Transcriptomic Analysis

To analyze transcriptomic data, Isl-1+ neonatal (N = 3) and adult (N = 3) pooled CPC gene transcripts were uploaded to Ingenuity Pathway Analysis (IPA) (Qiagen, Valencia, CA, USA). Core analysis was performed with specific parameters set for human cells. Categories specific to cancer and viral disease were removed and categories with a *p*-value < 0.05 were reported. IPA’s upstream regulator analysis tool was used to visualize significant networks generated from uploaded transcriptomic data. Lastly, transcripts were uploaded to StemChecker “http://stemchecker.sysbiolab.eu/as (accessed on 12 December 2021)” to determine the expression profile of the neonatal Isl-1+ CPC pool versus the adult Isl-1+ CPC pool. StemChecker refines the uploaded gene lists by identifying overlap with published literature in which stem cell gene sets (stemness signatures) are reported [24].

### 4.6. RT-PCR

RT-PCR was performed using the RT2 SYBR Green qPCR Master mix and the plate was loaded into the iQ5 RT-PCR Cycler. PCR plates were run under the following conditions: 94 °C for 10 min, 94 °C for 15 s, 58–60 °C (depending on primer) for 1 min, 72 °C for 30 s, repeated for 45 cycles. Primers for genes of interest are listed in Table 1 below. RT-PCR products were visualized using 1–2% agarose gel electrophoresis and a low mass DNA ladder was used to assess the size of the PCR product. Fold changes were calculated using the delta-delta Ct method [25].

### 4.7. Statistics

Data was processed using Microsoft Excel and GraphPad Prism 7.02. Transcriptomic data was used to generate heatmaps using GraphPad Prism 7.02.

## Figures and Tables

**Table 1:** Gene specific primer pairs (5′ to 3′).

Gene	Forward Sequence	Reverse Sequence
SALL4	CACAAGTGTCGGAGCAGTCT	CCGTCCGTACCTAACAGAGC
SOX2	AACCAGCGCATGGACAGTTA	GACTTGACCACCGAACCCAT
EpCAM	TGGGGAACAACTGGATCTGG	CCCACGCACACACATTTGTAA
TBX5	CTCAGTCCCCCGGAACAAC	CACGTACCTCCCAGCTCAAG
Tfap2c	TGGTTGGTTTTTGTGTCCGC	TTGCTTCGTGCCTACCCTTT

## Data Availability

The data presented in this study are openly available in FigShare under access number 10.6084/m9.figshare.21375648. Additional data can be made available upon reasonable request to the corresponding author.

## Abbreviations

CPC	Cardiac Progenitor Cell
Isl-1	Islet-1
SALL4	Spalt-like transcription factor 4
SOX2	SRY-Box Transcription Factor 2
EpCAM	Epithelial Cellular Adhesion Molecule
TBX5	T-box transcription factor 5
TFAP2C	Transcription Factor AP-2 Gamma
hESC	Human Embryonic Stem Cell
iPSC	Induced Pluripotent Stem Cell
RT-PCR	Reverse Transcription Polymerase Chain Reaction
IPA	Ingenuity Pathway Analysis
FHF	First Heart Field
SHF	Second Heart Field
